# Multivalvular involvement associated with Libman-Sacks endocarditis detected by multimodality imaging: A case report

**DOI:** 10.3389/fcvm.2023.1117711

**Published:** 2023-03-30

**Authors:** Son Tran Thanh Bui, Phuong Hoang Nguyen, Trang Ngoc Nguyen, James N. Kirkpatrick, Viet Khoi Nguyen, Hoai Thi Thu Nguyen

**Affiliations:** ^1^ Vietnam National Heart Institute, Bach Mai Hospital, Hanoi, Vietnam; ^2^ Allergy and Clinical Immunology Center, Bach Mai Hospital, Hanoi, Vietnam; ^3^ Radiology Center, Bach Mai Hospital, Hanoi, Vietnam; ^4^ Cardiovascular Division, Department of Medicine, University of Washington Medical Center, Seattle, United States; ^5^ Department of Internal Medicine, VNU—University of Medicine and Pharmacy, Hanoi, Vietnam

**Keywords:** Libman-Sacks endocarditis, three-dimensional echocardiography, cardiac magnetic resonance, cardiac computed tomography, multimodality imaging, nonbacterial thrombotic endocarditis, white matter hyperintensity

## Abstract

Libman-Sacks endocarditis accounts for 6–11 percent of systemic lupus erythematosus patients and is associated with varying degrees of valvular dysfunction, increased risk for stroke and transient ischemic attacks, and increased mortality. In previous studies, left-sided valvular Libman-Sacks vegetations were more frequently detected than right sided vegetations; reported cases of bilateral involvement is very rare. A comprehensive clinical assessment and the multimodality imaging is of utmost importance in the management of systemic lupus erythematosus. In this case report, we describe a 31-year-old female patient with uncontrolled systemic lupus erythematosus initially presented with gastrointestinal symptoms but eventually had a vegetation-like structure on the posterior leaflet of the mitral valve which was revealed during routine echocardiography. Two-dimensional/three-dimensional transthoracic and transesophageal echocardiography, cardiac magnetic resonance, and cardiac computed tomography further characterized the mitral valve vegetation and revealed an additional vegetation of the pulmonary valve. Echocardiography remains the cornerstone for the detection of Libman-Sacks vegetations. Cardiac MRI and cardiac CT are useful in characterizing lesion size and effects and may prove particularly helpful in the assessment of right-sided or multivalvular endocarditis. The presence of focal brain lesions on brain MRI prompted antithrombotic therapy.

## Introduction

Libman-Sacks endocarditis (LSE) is a form of nonbacterial thrombotic endocarditis (NBTE) and is found in 6%–11% of patients with systemic lupus erythematosus (SLE) (1). Most LSE cases are clinically silent. However, the presence of Libman-Sacks vegetations is associated with varying degrees of valvular dysfunction, increased risk for stroke and transient ischemic attacks, and increased mortality (1, 2). Early detection and initiation of medical therapy may resolve LSE and prevent valvular deterioration (3). In previous studies, left-sided valvular Libman-Sacks vegetations were more frequently detected than right sided vegetations; reported cases of bilateral involvement is very rare (1). In this case report, we highlight the importance of multimodality imaging in the diagnosis of multivalvular involvement in LSE. Our case report was presented in line with the CARE criteria (4).

## Case presentation

A 31-year-old woman diagnosed with SLE 12 years prior presented to our immunology clinic. She complained of a one-month history of general swelling of the whole body and diarrhea. Her SLE was not well-controlled and was predominantly hematological during her previous flares. She had a prior history of intolerance to hydroxychloroquine and was taking methylprednisolone and mycophenolate mofetil at the time of admission. She was positive for SARS-CoV-2 one month prior to admission without any symptom. Her review of systems was negative for underlying valvular disease, previous episodes of rheumatic fever, antiphospholipid syndrome (APS), and intravenous drug abuse.

On examination, the patient had normal vitals, was afebrile, and had generalized edema. A thorough cardiovascular evaluation was unremarkable with no cardiac murmurs and normal jugular venous pressure. Examination of the integumentary system did not show any purpura, splinter hemorrhage, oral ulcers, or alopecia. Notable initial laboratory findings were as follows: mild normocytic anemia with negative Coombs tests, thrombocytopenia (45 × 10^9^ per liter), leukocytosis (17.12 × 10^9^ per liter), decreased C3 levels (0.45 g/L), severe hypoalbuminemia (8.6 mg/L) without evidence of proteinuria or hematuria, within-normal range liver and renal function tests. Her procalcitonin level was mildly elevated (0.166 ng/ml). The C-reactive protein, interleukin-6, high-sensitivity cardiac troponin and N-type brain natriuretic peptide were in normal ranges. Her coagulation studies showed a prothrombin time and fibrinogen level within normal limits but mildly elevated D-dimer level (0.95 mg/L). The immunologic study was positive for antinuclear antibodies and anti-double-stranded DNA. Screening for antiphospholipid (aPL) antibodies was negative. Endoscopic evaluation of the upper and lower gastrointestinal tract with biopsies only revealed lymphoplasmacytic infiltrates without any evidence of celiac disease. Her SLEDAI-2K score was 5.

**Figure 1:**
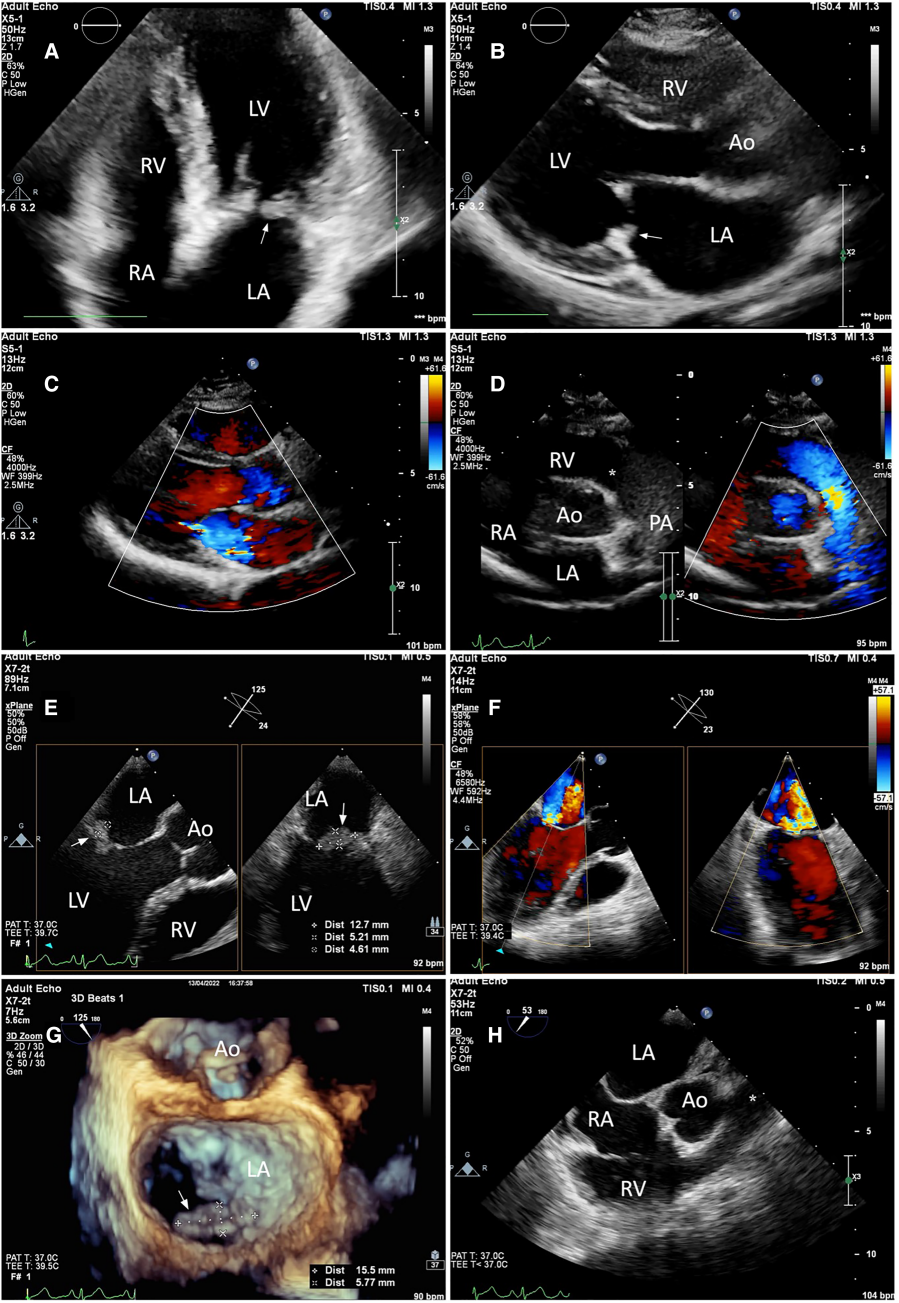
Still-frame images of the mitral and pulmonary valves on TTE (**A–D**) and TEE (**E–H**). (**A,B**) TTE apical four-chamber and parasternal long axis views revealed a well-circumscribed, sessile mass with heterogeneous echotexture which was firmly attached to the atrial side of the posterior leaflet of the mitral valve (white arrow). The mitral valve leaflets were thickened. (**E**) The maximum diameter of the mass was measured using 2D Xplane imaging at the mid-esophageal level. (**G**) From the left atrial perspective (en-face view), the vegetation was seen attached to the atrial side of the P2 and P3 portions of the posterior leaflet (white arrow) with 3-dimensional zoom-mode. (**C,F**) mild-to-moderate central mitral regurgitation was seen on both TTE and TEE with color Doppler. (**D,H**) The pulmonary valve as visualized on the parasternal short-axis and mid-esophageal right ventricle inflow-outflow view was unremarkable (asterisk). Annotations: LA, left atrium; LV, left ventricle; RA, right atrium; RV, right ventricle; Ao, Aorta; PA, pulmonary artery.

A transthoracic echocardiogram (TTE) revealed a 6-by-13-millimeter mass of heterogeneous echocardiographic texture which was well-circumscribed, oval-shaped, protruding, sessile, and firmly attached to the atrial side of the P2 and P3 portions of the posterior mitral leaflet. The posterior mitral leaflet was diffusely thickened, predominantly at the tip and mid portions. There was no commissural fusion, calcification of the leaflets, or involvement of the subvalvular apparatus. Mild-to-moderate central mitral regurgitation and trivial pericardial effusion were present. To further elucidate the nature of the mass and rule out possible thrombi, 2-dimensional and 3-dimensional transesophageal echocardiography (TEE) was subsequently performed (Figures 1E–H). The other valves were morphologically normal. Left ventricular systolic function was preserved with a left ventricular ejection fraction (LVEF) of 74 percent. Two-dimensional left ventricular and right ventricular global longitudinal strain values were normal.

**Figure 2:**
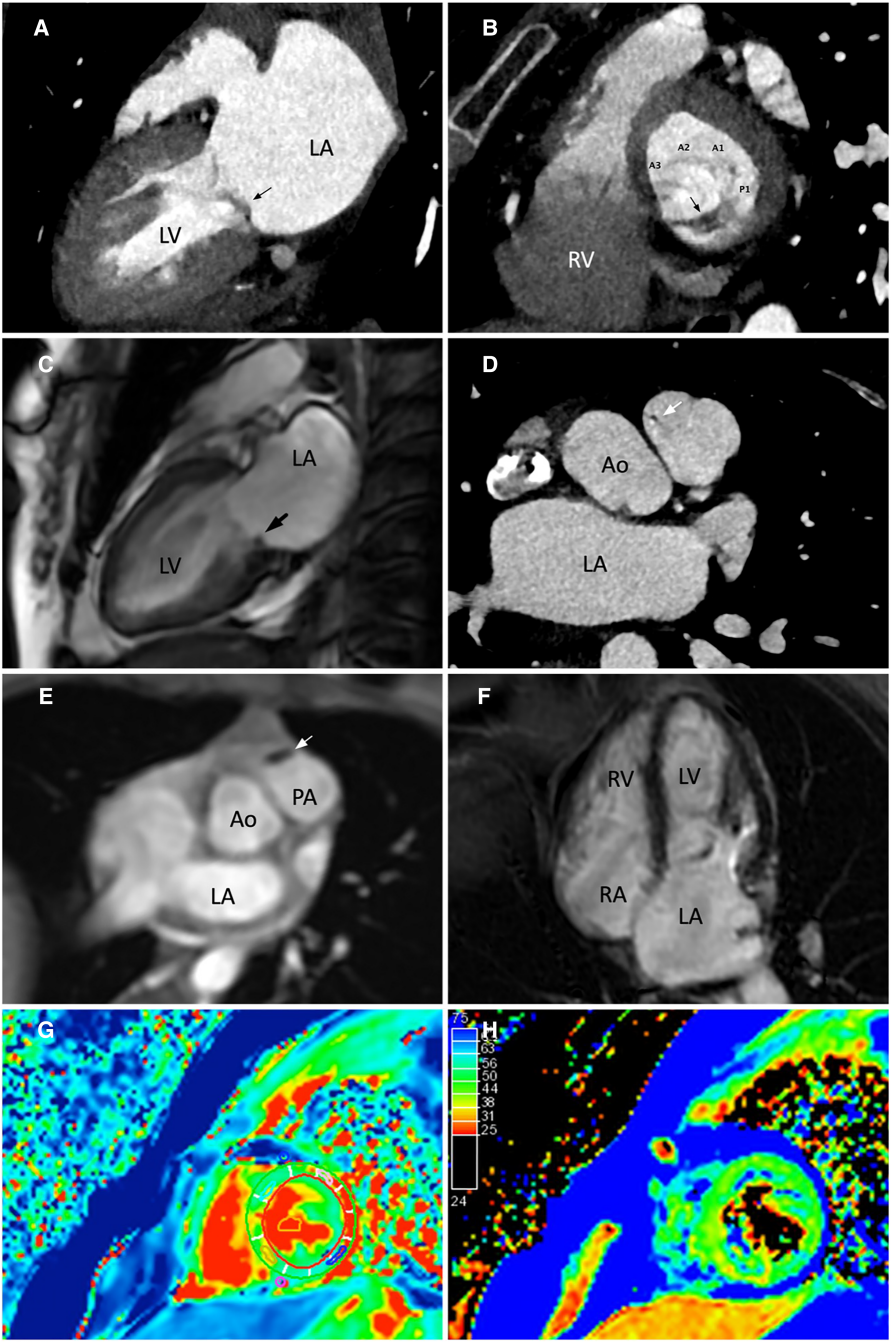
Evaluation of the mitral and pulmonary valve vegetations using cardiac computed tomography (CT) and cardiac magnetic resonance (CMR). (**A,C**) CT 2-chamber view and cine-CMR still image in end-systole showing a non-homogeneous, isodense mass involving the posterior leaflet of the mitral valve. (**B**) CT short axis view of the mitral valve from a basal short axis slice demonstrated the attachment of the vegetation to the P2 and P3 portions of the posterior mitral leaflet. The mitral valve was seen from a left ventricular perspective. (**D,E**) Transverse CT and CMR images through the right ventricular outflow tract showed a small oscillating mass on the anterior cusp of the pulmonary valve. (**F**) The myocardium showed no late gadolinium enhancement. (**G,H**) Normal findings on native-T1 and T2-weighted mapping images. Annotations: LA, left atrium; LV, left ventricle; RA, right atrium; RV, right ventricle; Ao, Aorta.

**Figure 3:**
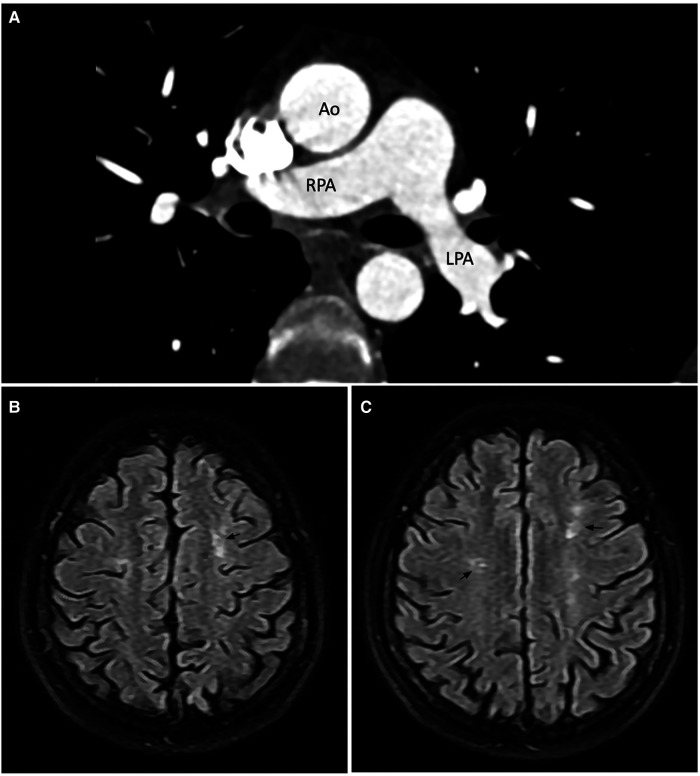
Computed tomography pulmonary angiogram (**A**) and brain magnetic resonance imaging scan (**B,C**). (**A**) No large pulmonary emboli on CTPA. **(B,C)** Axial FLAIR image showed bilateral hyperintense white matter lesions in the frontal lobes. The number of lesions is more than expected for the patient's age. Annotations: Ao, aorta; RPA, right pulmonary artery; LPA, left pulmonary artery.

A cardiac magnetic resonance (CMR) demonstrated a preserved LVEF (60.5%) and no regional wall motion abnormalities. Late gadolinium enhancement (LGE) images, native-T1 map, T2 map, and extracellular volume (ECV) map were normal (Figures 2C–H). Hence, lupus-induced myocarditis was excluded. The mitral valve vegetation appeared as a non-homogeneous, isodense mass involving the posterior leaflet. Transverse cine images through the right ventricular outflow tract revealed a 1.4-by-2.5-millimeter oscillating mass on the anterior cusp of the pulmonary valve which was not seen on echocardiography. Electrocardiogram-gated cardiac computed tomography (CT) with multiplanar reconstruction was subsequently performed to better delineate the exact dimensions of the vegetations (Figures 2A,B). Obstructive lesions were not found on her coronary CT angiography. CT of the abdomen and the pulmonary vasculature was negative for emboli (Figure 3A) and possible neoplasms. Deep bilateral white matter hyperintensities (WMH), which were more extensive than expected for the patient's age, were found on her brain magnetic resonance imaging (MRI) scan (Figures 3B,C).

A comprehensive investigation for possible infective endocarditis (IE) including three sets of blood cultures, serologic testing for fastidious organisms, and stool sample culture did not reveal any pathogens. The final diagnosis was concomitant mitral and pulmonary valve LSE in a patient with lupus flare and protein-losing enteropathy. The patient was treated with methylprednisolone, systemic anticoagulation with subcutaneous enoxaparin, and intravenous ceftriaxone. After one month of medical therapy, the clinical and laboratory profile improved with a SLEDAI-2K score of 2. A follow-up pre-discharge 3D-TEE showed an unchanged size of the vegetation and severity of mitral regurgitation as compared to earlier echocardiographic studies.

**Figure 4:**
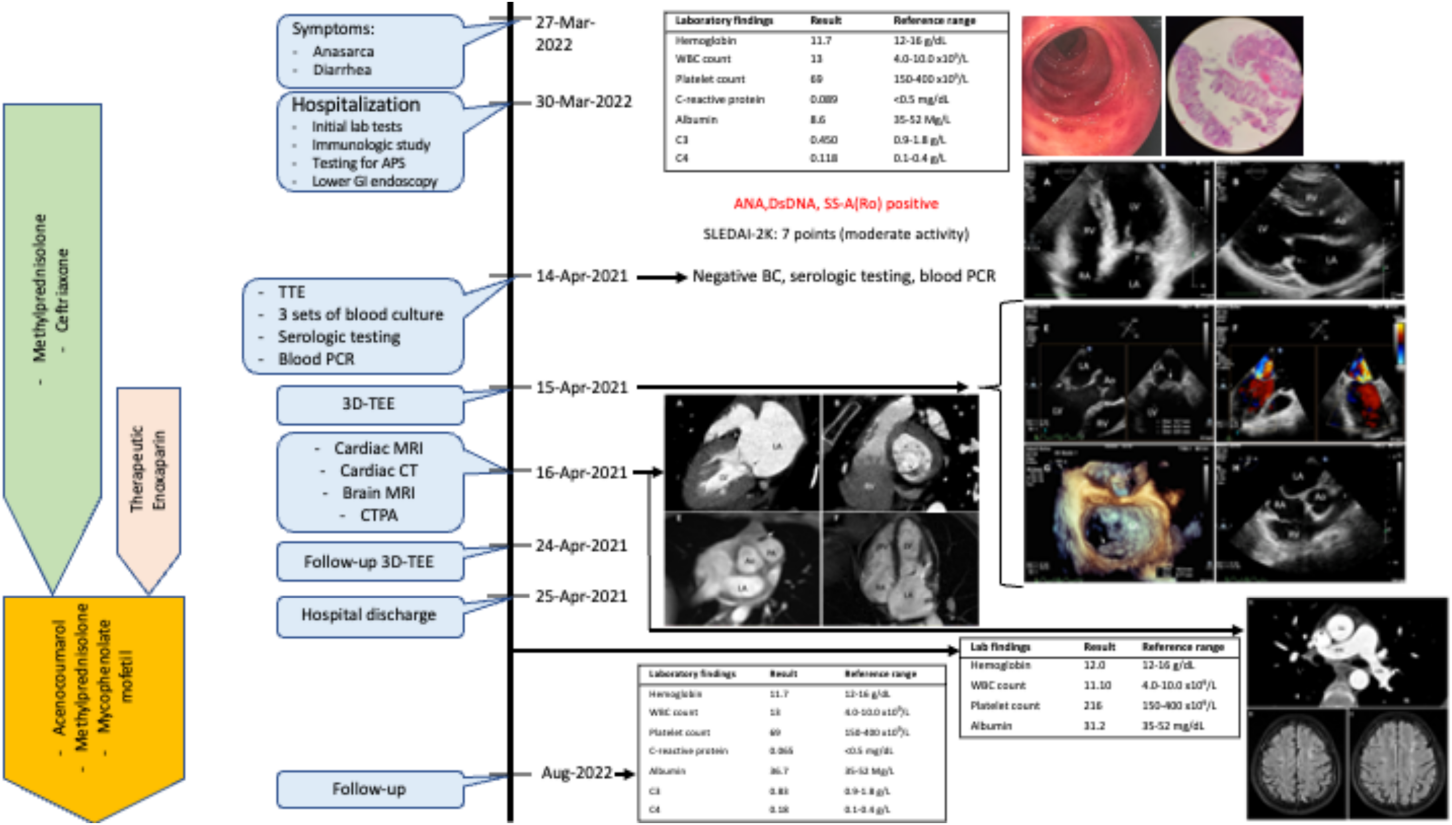
Timeline of patient clinical course.

After discharge, the patient maintained long-term therapeutic anticoagulation with acenocoumarol in addition to tapered-dose oral methylprednisolone and mycophenolate mofetil. At 3-month follow-up, she reported total remission of symptoms, and her laboratory tests demonstrated well-controlled SLE. Nonetheless, the Libman-Sacks vegetations and moderate mitral regurgitation persisted on subsequent TTEs. If our patient remains asymptomatic, repeated subsequent TTEs are planned every 1–2 years. The timeline of the patient clinical course is shown in Figure 4.

## Discussion

The true prevalence of LSE can reach 50% in autopsy studies (1). LSE is associated with patients having longer lupus duration, higher disease activity, anticardiolipin antibodies, and antiphospholipid syndrome (1). Hence, these populations may warrant screening for LSE, even if asymptomatic.

Despite its clinical significance, LSE is often overlooked during routine echocardiography. The sensitivity of TTE in the detection of LSE is only 11% (1). Most LSE cases are asymptomatic, and a prior SLE diagnosis can also bias echocardiogram interpretations (5). Aside from being more sensitive and specific (6), TEE allows a better understanding of the mechanism of valvular diseases and rules out cardiac thrombi. 3D-TEE has incremental value over 2D-TEE for the detection and characterization of vegetations.

Echocardiography remains the cornerstone for the assessment of cardiac vegetations because it has unrivaled temporal resolution and repeatability. However, it is operator-dependent and is sometimes hindered by body morphology and artifacts from surrounding tissues. Furthermore, due to the complex anatomy and anterior position of the right heart, visualization of the right-sided valves by conventional 2D-TTE and TEE is not optimal, which may explain left-sided predominance of LSE in previous reports. The advent of novel cardiac imaging modalities addresses these difficulties.

CMR is effective in the diagnosis of LSE through direct visualization of vegetations on cine-CMR (steady-state free precession). CMR phase contrast imaging is an appealing new method to quantify regurgitation severity because of its ability to directly measure the flow across a valve. Myocardial tissue characterization using LGE analysis, T1, T2, ECV mapping, and perfusion imaging can identify patients with silent lupus-induced myocardial and pericardial disease (7). Because SLE affects any parts of the heart, CMR is the preferred imaging modality in asymptomatic or oligosymptomatic cases especially when echocardiographic features are abnormal (7). However, vegetations smaller than 3 millimeters are difficult to visualize on CMR due to inferior spatial resolution and partial volume artifact (8).

In comparison with CMR, ECG-gated CCT has a spatial resolution of 0.5–1 millimeters which is excellent at evaluating small structures (9). Nevertheless, it has a lower temporal resolution and requires exposure to radiation and nephrotoxic contrast agents. In our case, the improved spatial resolution allowed better localization and sizing of the pulmonary vegetation previously seen on cine-CMR.

Several conditions to be considered in the differential diagnosis include IE, papillary fibroelastoma, Lambl's excrescences, and cancer-related NBTE. Infective vegetations are often independently highly mobile, elongated, have narrow attachment to its base, and may accompany valve perforation (10). Fibroelastoma rarely causes valvular dysfunction and tends to localize away from the leaflets' free edge (8). Lambls's excrescences appear on echocardiography as undulating hypermobile, strand-like echodensities at the leaflets' coaptation (2). A primary neoplasm was not evident on CT and MRI scans of the thorax, abdomen, and brain. The absence of all forementioned features was ascertained using multimodality imaging.

Treatment mainly involves immunosuppressive therapy for underlying lupus and systemic anticoagulation (11). Surgery in LSE is associated with high mortality and should be reserved only for patients having severe valvular dysfunction, very large vegetations (greater than 2 centimeters), or recurrent thromboembolism despite therapeutic anticoagulation, after weighing the benefits and risks of surgery (3, 11). LSE is associated with cerebrovascular embolism, focal brain lesions, and neuropsychiatric involvement (1, 2). Conversely, patients who exhibit deep WMH on brain MRI scans have increased risk for ischemic stroke (12). Early anti-inflammatory and anti-thrombotic therapy might resolve vegetations-induced valvular dysfunction, improve cerebral perfusion, and avoid the need for surgery (3).

As LSE is highly prevalent among SLE patients with aPL antibodies, most data guiding the choice of antithrombotic agents in LSE was derived from the experience in managing thromboembolic APS. Therapeutic anticoagulation with rivaroxaban in high-risk APS patients (i.e., having triple positive aPL) showed increased risk of recurrent thromboembolic events as compared to conventional vitamin K agonists (VKAs) (13). The 2019 European Society of Cardiology guidelines recommend against the use of DOACs in all APS patients (14). The 2020 International Society on Thrombosis and Haemostasis guidance provided more detailed indications stating that warfarin should be the first-choice treatment in APS with arterial thromboembolic events, triple positivity, small vessel thrombosis, or valvular disease (including LSE), and that DOACs should only be considered in venous APS with single or double positivity or patients who cannot tolerate or have contraindications to warfarin (15). Mantovani et al. reported the recurrence of thromboembolic events in cancer-related NBTE patients while under therapeutic DOAC (16). Because both cancer-related and auto-immune mediated NBTE share the same spectrum of histopathologic lesions and are associated with hypercoagulable states (17), the use of DOACs in LSE should be cautioned. Although more high-quality trials are needed, available data and guidelines support the use of VKAs in aPL-negative LSE (like our patient). Patients having recurrent thromboembolic events while on oral anticoagulants should be switched to heparin.

## Conclusion

LSE can involve multiple heart valves. Echocardiography remains the cornerstone for its detection, but cardiac MRI and cardiac CT can be useful in characterizing lesion size and effects and may prove particularly helpful in the assessment of right-sided or multivalvular Libman-Sacks endocarditis. The presence of focal brain lesions can guide antithrombotic therapy in asymptomatic, uncomplicated LSE.

## Data Availability

The original contributions presented in the study are included in the article/Supplementary Materials, further inquiries can be directed to the corresponding author.
